# First Report of a Mesonivirus and Its Derived Small RNAs in an Aphid Species *Aphis citricidus* (Hemiptera: Aphididae), Implying Viral Infection Activity

**DOI:** 10.1093/jisesa/ieaa022

**Published:** 2020-04-13

**Authors:** Tengyu Chang, Mengmeng Guo, Wei Zhang, Jinzhi Niu, Jin-Jun Wang

**Affiliations:** 1 College of Plant Protection, Key Laboratory of Entomology and Pest Control Engineering, Southwest University, Chongqing, China; 2 Academy of Agricultural Sciences, International Joint Laboratory on China-Belgium Sustainable Crop Pest Control, Southwest University, Chongqing, China

**Keywords:** Nidovirus, Mesonivirus, RNA interference, mall RNA

## Abstract

We report a new positive-sense single-stranded RNA (ss RNA+) virus from the brown citrus aphid *Aphis citricidus*. The 20,300 nucleotide (nt)-long viral genome contains five open-reading frames and encodes six conserved domains (TM2, 3CLpro, TM3, RdRp, Zm, and HEL1). Phylogenetic analysis and amino acid sequence analysis revealed this virus might belong to an unassigned genus in the family Mesoniviridae. The presence of the virus was also confirmed in the field population. Importantly, analysis of the virus-derived small RNAs showed a 22-nt peak, implying that viral infection triggers the small interfering RNA pathway as antiviral immunity in aphids. This is the first report of a mesonivirus in invertebrates other than mosquitoes.

The order Nidovirales are positive-sense single-stranded RNA (ssRNA+) viruses including nine families (e.g., Arteriviridae, Coronaviridae, Euroniviridae, and Medioniviridae) (Siddell et al. 2019). The International Committee on Taxonomy of Viruses listed the newly established Mesoniviridae as a new member of Nidovirales (Zirkel et al. 2013), which is based on two closely related viruses, Cavally virus (Zirkel et al. 2011) and Nam Dinh virus (Nga et al. 2011). Mesoniviridae comprises nine viruses, which were isolated from mosquitoes (Zhou et al. 2017). Nidoviruses normally have conserved genomic composition and replication strategies and contain multiple open-reading frames (ORFs). The 5′ region of the genome encodes two partially overlapping large ORFs, namely ORF1a and ORF1b, which translate polyprotein 1a (pp1a) and polyprotein 1ab (pp1ab), respectively. The ORF1a-encoded protease hydrolyzes the pp1a and pp1ab proteins to produce a variety of products that regulate genomic expression and replication. The 3C-like protease surrounded by the transmembrane domain is encoded by ORF1a. The RNA-dependent RNA polymerase and superfamily 1 helicase (HEL1) are encoded by ORF1b. The 3′ region of the genome of order Nidovirales includes several smaller ORFs, while their number varies among viruses (Gorbalenya et al. 2006) and may encode structural proteins. Recently, some novel aphid viruses, such as *Aphid lethal paralysis virus* (Van Munster et al. 2002), *Brevicoryne brassicae virus* (Ryabov 2007), *Rosy apple aphid virus* (Ryabov et al. 2009), *Aphis glycines virus 2* (Liu et al. 2016), *Aphis glycines virus 1* (Yasmin et al. 2020), and *Aphis citricidus bunyavirus* in brown citrus aphid, *Aphis* (formerly *Toxoptera*) *citricidus* (Zhang et al. 2019), were reported through RNA-seq. In this study, we report a novel virus belonging to Nidovirales in an aphids species, brown citrus aphids, preliminarily named as *Aphis citricidus* meson-like virus (AcMSV). On further analyses of bases on virus-derived small RNAs, we found 22-nt peak of AcMSV, implying that the infection of this virus triggers the antiviral immunity of the host aphids that infest on Rutaceae plants and is the vector of *Citrus triseza virus* (Hunter et al. 2003). Our study will aid in enhancing our knowledge on the host range of mesonivirus, besides, the discovery of this virus may also facilitate our understanding of the aphids ecology and aphids pest control approach.

## Materials and Methods

### Insects

Aphid species were collected from citrus trees in the greenhouse of Southwest University, Chongqing, China in 2012 and were raised to date in a chamber of artificial climate at 60%−65% RH (relative humidity), with a 14:10 (light:dark) h photoperiod (Shang et al. 2015).

### Next-Generation Sequencing

Twenty adults and 20 nymphs were selected and their total RNA was isolated using the TRIzol reagent (Invitrogen, Carlsbad, CA). From total RNA, two libraries were constructed: i) an RNA-seq library to find possible RNA virus sequences. The Ribo-zero Magnetic Kit (Epicenter, Madison, WI) was used to remove rRNA from the total RNA. Next, the TruSeq Total RNA sample preparation kit (Illumina, San Diego, CA) was used to construct the RNA-seq library and a HiSeq X Ten platform with PE 150 bp was used for sequencing to generate ~8 Gbp of original data. ii) A small RNA library for analyzing small RNAs from potential viruses. The redundant overlapping sequences were removed to obtain a unique sequence.

### Sequence Confirmation

For full genome sequencing, we designed nine pairs of primers to obtain nine fragments (1,900−3,400 nt) by RT–PCR for sequence confirmation, and each fragment had a 100−300 nt overlap with other fragments (Supp Fig. S1 and Table 1 [online only]).

### Phylogenetic and Sequence Analysis

The ORFs were determined using Gene Finding in Viral Genomes (http://linux1.softberry.com/berry.phtml?topic=virus0&group=programs&subgroup=gfindv). The isoelectric point and molecular weight were predicted by ExPASy (https://web.expasy.org/compute_pi/). The presence of conserved protein domains in *Aphis citricidus* meson-like virus was determined using the SMART tool (http://smart.embl-heidelberg.de/smart/set_mode.cgi?GENOMIC=1) and the Conserved Domain Database (CDD) in NCBI (https://www.ncbi.nlm.nih.gov/Structure/cdd/cdd.shtml). The neighbor-joining (NJ) phylogenetic tree was constructed using MEGA5.1 based on the amino acid alignment of the highly conserved domain RNA-dependent RNA polymerase. Analyses of the deduced aa homologies of the three conserved protein domains and two ORFs of AcMSV and other representative mesoniviruses were carried out using the DNAstar MEGalign tool. The pairwise evolutionary distance (PED) was used to further determine the species boundary criteria for AcMSV in the family Mesoniviridae (Lauber and Gorbalenya 2012). When the PED between each strain is greater than 0.032, the strain can be divided into different species.

### Virus Detection and Quantification

To determine the viral existence in the field population of *A. citricidus* and the rate of AcMSV infection among different geographic populations, we collected two field populations of *A. citricidus* from different areas for virus detection. The two field populations were collected from citrus trees in Beibei District of Chongqing (106.38°E, 29.77°N) and Huaxi District of Guiyang (106.67°E, 26.51°N) on 18 October 2019, and 11 December 2019, respectively. From each population, 20 adult brown citrus aphids were selected (5 aphids per citrus tree and 4 replicates), and RNA from a single aphid sample was used to synthesize cDNA. A pair of primers was designed based on the genomic sequence of AcMSV to amplify an 1,101-bp fragment (Supp Fig. S2 and Table 1 [online only]). The PCR was performed as follows: 35 cycles at 94°C for 30 s, 60°C for 30 s, and 72°C for 70 s. Amplification was observed through 1% agarose gel electrophoresis. Different tissues of the laboratory population of brown citrus aphids were dissected to analyze the AcMSV distribution. The viral load of AcMSV was quantified by carrying out qPCR using the Bio-Rad CFX384 Real-Time Detection System (Bio-Rad, USA) with NovoStart SYBR qPCR SuperMix Plus (Novoprotein, China). Thermal cycling conditions were: 95°C for 180 s, 40 cycles of 95°C for 30 s, and 60°C for 30 s. Four replicates of the reaction of samples from each group were performed. Reference genes, *EF1α*, and *β-act*, were used to normalize the expression of AcMSV by qBASE.

## Results

**Fig. 1.**
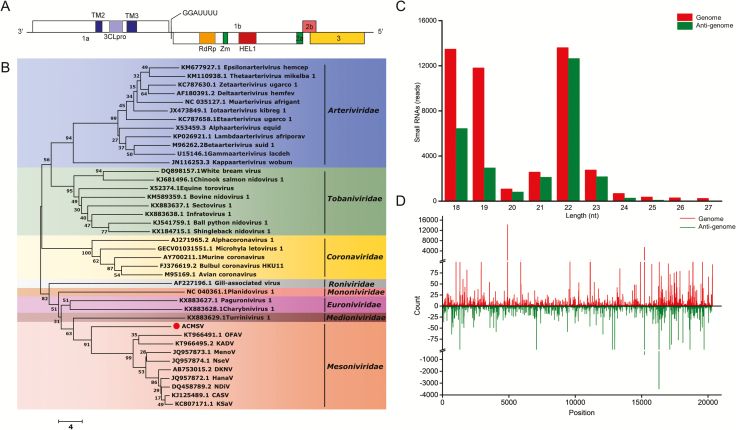
(A) Genome organization of *Aphis citricidus* meson-like virus. 3CLpro, 3c-like protease; RdRp, RNA-dependent RNA polymerase; Zm, zinc cluster-binding domain fused to HEL1; HEL1, the superfamily 1 helicase. (B) Phylogenetic analysis of *Aphis citricidus* meson-like virus based on the amino acid sequence of RdRp domains. The phylogenetic tree was constructed by the NJ method with 1,000 bootstrap replications. (C) The read-length distribution of *Aphis citricidus* meson-like virus-derived small RNAs. (D) The distribution of ~22-nt-long small RNAs (vsiRNA) in *Aphis citricidus* meson-like virus genome and antigenome regions. The red lines represent the counts of vsiRNAs from the *Aphis citricidus* meson-like virus genome, and the green lines represent the count from antigenome.

This virus contains a positive sense, single-stranded RNA genome; besides the 5′ ends, the genome is 20,300 nt in length (Fig. 1A). The virus contains partial 5′-untranslated region (UTR) (281 nt), 3′UTR (19,731−20,300 nt), and 5 ORFs including ORF1a (282−8258 nt), ORF1b (8516−15,769 nt), ORF2a (15,766−16,125 nt), ORF2b (16,118−16,900 nt), and ORF3 (16,551−19,730 nt). The molecular weights of these five ORFs are, respectively, 308.1, 281.3, 14.2, 29.7, and 121.7 kDa. Unlike the NDiV, where the smooth sequence GGAUUUU is located at ORF1a/ORF1b overlap region and controls ORF1a/ORF1b 1 ribosomal frameshift (RFS) (Nga et al. 2011), the ‘slippery sequence’ GGAUUUU of AcMSV is between ORF1a and ORF1b is located between ORF1a and ORF1b of AcMSV. As per the NCBI Conserved Domain Database and SMART analysis, the virus contains six conserved domains, including TM2, 3CLpro, TM3, RdRp, Zm, and HEL1 (Fig. 1A). TM2, 3CLpro, and TM3 are encoded by ORF1a, and RdRp, Zm, and HEL1 are encoded by ORF1b. TM2 and TM3 are transmembrane domains, 3CLpro is a 3c-like protease, RdRp is an RNA-dependent RNA polymerase, and Zm is a zinc cluster-binding domain fused to HEL1 (Gorbalenya et al. 2006).

A phylogenetic tree of AcMSV was constructed based on the conserved domain of RdRp using the NJ method (Fig. 1B). The deduced amino acid sequence of the putative RdRp gene of AcMSV was aligned with that of major nidoviruses. While the RdRp sequence was found to be clustered in the family Mesoniviridae, AcMSV was separated from the other nine mesoniviruses. Sequence alignments show that the similarity between AcMSV and other representative mesoniviruses is lower than the similarity between other mesoniviruses (Supp Table 2 [online only]). Furthermore, the superfamily 1 helicase (Hel1) domain, which is conserved in all nidoviruses, has a higher identity than other protein domains. We calculated the PED between AcMSV and the nine previously described mesoniviruses (CASV, HanaV, MenoV, KSaV, NDiV, OFAV, KADV, and NseV) using ORF1b (Supp Table 3 [online only]). The results reveal that the PED between ORF1b of each virus was significantly higher than the recommended cutoff value of 0.032 to delineate species boundaries. The PED between the AcMSV virus and the other nine mesoniviruses was significantly higher than that among other mesoniviruses. These results, therefore, provide evidence that AcMSV should be grouped in a new virus genus.

The small interfering RNA pathway refers to the process of degradation of viral genomes by identifying viral dsRNA and then the production of 21- to 23-nt viral-derived small interfering RNAs, which in turn recognize viral RNA via complimentary binding (Bronkhorst and van Rij 2014). Therefore, studies on virus-derived small RNA (vsRNA) from AcMSV can demonstrate the infectious activity of the virus in *A. citricidus* concomitantly reflecting the immune activity of the host against the virus. A large number of typical 22-nt vsRNA peaks was observed on the bioinformatic analysis of small RNA libraries (Fig. 1C). On mapping vsRNAs back to the AcMSV genome, the proportion of the vsRNA sequences of the positive and negative strands of AcMSV was found to be symmetrically distributed (Fig. 1D), indicating the activation of the Dicer protein in the small interfering RNA pathway of the brown citrus aphid and processing of the viral-associated dsRNA into mainly 22bp vsRNA, thus providing an antiviral mechanism to protect the host.

The infection rates of AcMSV in *A. citricidus* samples from the laboratory population, and two field populations (Chongqing and Guizhou) were analyzed. In total, eight positive samples were detected from laboratory population (*n* = 20) (Supp Fig. S2A [online only]), ten positive samples were detected from Chongqing population (*n* = 20) (Supp Fig. S2B [online only]), and no positive samples were detected from Guizhou population (*n* = 20) (Supp Fig. S2C [online only]). Significant variation in viral titer in different tissues was seen in muscle, wing, CNS, embryo, fat body, integument, gut, and salivary gland of alate (*F*[7, 24] = 14.194, *P* = 0.000). The highest viral titer was found in salivary glands in alate morph (Supp Fig. S3A [online only]). The viral titers were also significantly different in CNS, embryo, fat body, integument, gut, and salivary glands of apterous morph (*F*[5, 18] = 12.006, *P* = 0.000), while the highest viral titer was observed in the gut (Supp Fig. S3B [online only]).

## Discussion

In this study, we discovered a novel aphid virus AcMSV. This virus was classified as a member of the order Nidovirales based on its genomic structure, putative proteome characteristics, amino acid sequence identity, PED, and phylogenetic analysis. In phylogenetic analysis, although relatively distant, AcMSV clustered with viruses of the family Mesoniviridae. This new report expands our knowledge on the host range of mesonivirus, which was previously reported only in mosquitos. In addition, based on vsRNA profiles of AcMSV, it seems that AcMSV not only infects the aphids but also triggers the small interfering RNA pathway as host antiviral immunity. During viral infection, virus-related dsRNAs are generated, such as replication intermediates, dsRNA viral genome itself, virus-encoded siRNAs, and viral transcript-genome hybrids. These viral related dsRNAs were processed by Dicer-2, and then viral derived siRNAs (vsiRNAs) were generated symmetrically from the viral genome and the anti-genome. The size of vsiRNAs differs in different insects (Santos et al. 2019). In aphids, the size of vsiRNAs seems to be 22 nt, arising symmetrically from the viral genome and anti-genome. However, the peak of 18- and 19-nt RNAs was not symmetrical from viral genome and anti-genome, indicating that they may not be vsiRNAs. Although it is difficult to conclude the origin and function of these vsiRNAs, it seems that the virus can also produce certain small RNAs in regulating host–virus interactions (Asgari 2018). The test results showed that only about half of the laboratory aphids were AcMSV positive, which may be due to the parthenogenesis of aphids under laboratory feeding conditions. The limited communication between the populations did not make the aphid infection rate particularly high. Furthermore, the sensitivity of PCR detection is limited, and may not detect when the virus content is small. The presence of the virus was also confirmed in one field population, indicating potential ecological interactions between virus and aphids in the field. Intriguingly, titer profiles of AcMSV showed a different pattern in alate and apterous morphs which still require further investigation. Although we did not observe any pathogenicity in the brown citrus aphid upon the presence of AcMSV in our lab conditions, further studies are required to elucidate the virus–host interaction between AcMSV and aphids in enhancing our understanding of the ecology of aphids.

## Supplementary Material

ieaa022_suppl_Supplementary_Material
