# Supplementary material for: First Report of a Mesonivirus and Its Derived Small RNAs in an Aphid Species Aphis citricidus (Hemiptera: Aphididae), Implying Viral Infection Activity
Source: J Insect Sci. 2020 Apr 13;20(2):14. doi: 10.1093/jisesa/ieaa022 (PMC7153580; doi:10.1093/jisesa/ieaa022)
Supplement: ieaa022_suppl_Supplementary_Material [file ieaa022_suppl_supplementary_material.docx]

Jin-Jun Wang

College of Plant Protection

Southwest University

Chongqing, 400715, China

Phone: +86 (138) 8338-1770

E-mail: [wangjinjun@swu.edu.cn](mailto:wangjinjun@swu.edu.cn)

**First report of a** **mesonivirus and its derived small RNAs in** **an aphid** **species *Aphis citricidus* (Hemiptera: Aphididae), implying viral infection activity**

Tengyu Chang ^1,2^ , Mengmeng Guo ^1,2^ , Wei Zhang ^1,2^ , Jinzhi Niu^1,2^ , Jin-Jun Wang^1,2,3^

^1^College of Plant Protection, Key Laboratory of Entomology and Pest Control Engineering, Southwest University, Chongqing 400715, China, ^2^Academy of Agricultural Sciences, International Joint Laboratory on China‑Belgium Sustainable Crop Pest Control, Southwest University, Chongqing 400715, China, and ^3^Corresponding author; e-mail: [wangjinjun@swu.edu.cn](mailto:wangjinjun@swu.edu.cn)


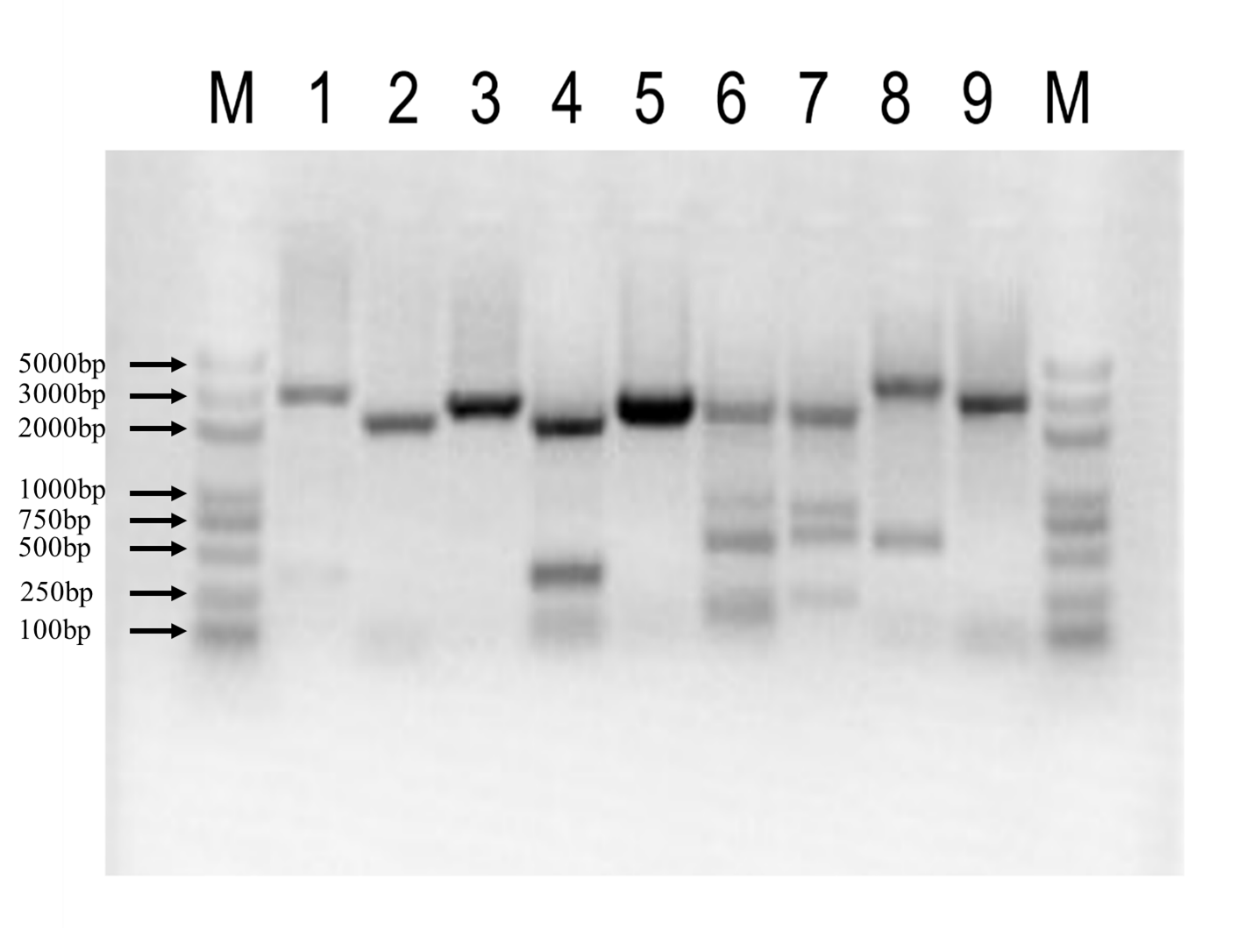


**Fig. S1.** RT-PCR and agarose gel electrophoresis to confirm the virus sequence. Lanes 1 to 9 are the nine parts derived from virus sequences. M: BM5000 marker.

**
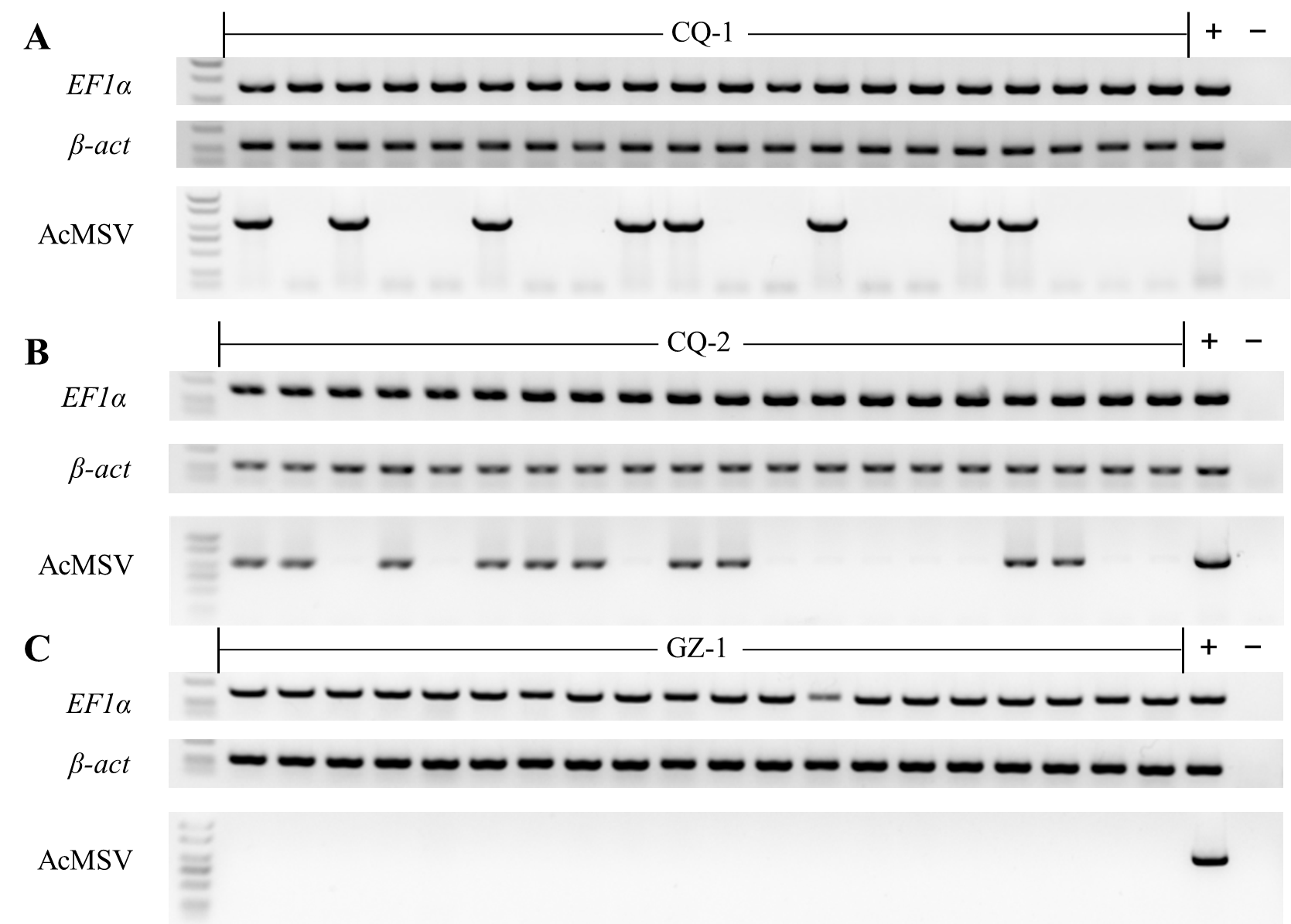
**

**Fig.S2.** Detection of AcMSV in different populations (A: Laboratory population originally collected in Chongqing; B: filed population collected in Chongqing; C: filed population collected in Guizhou).

**
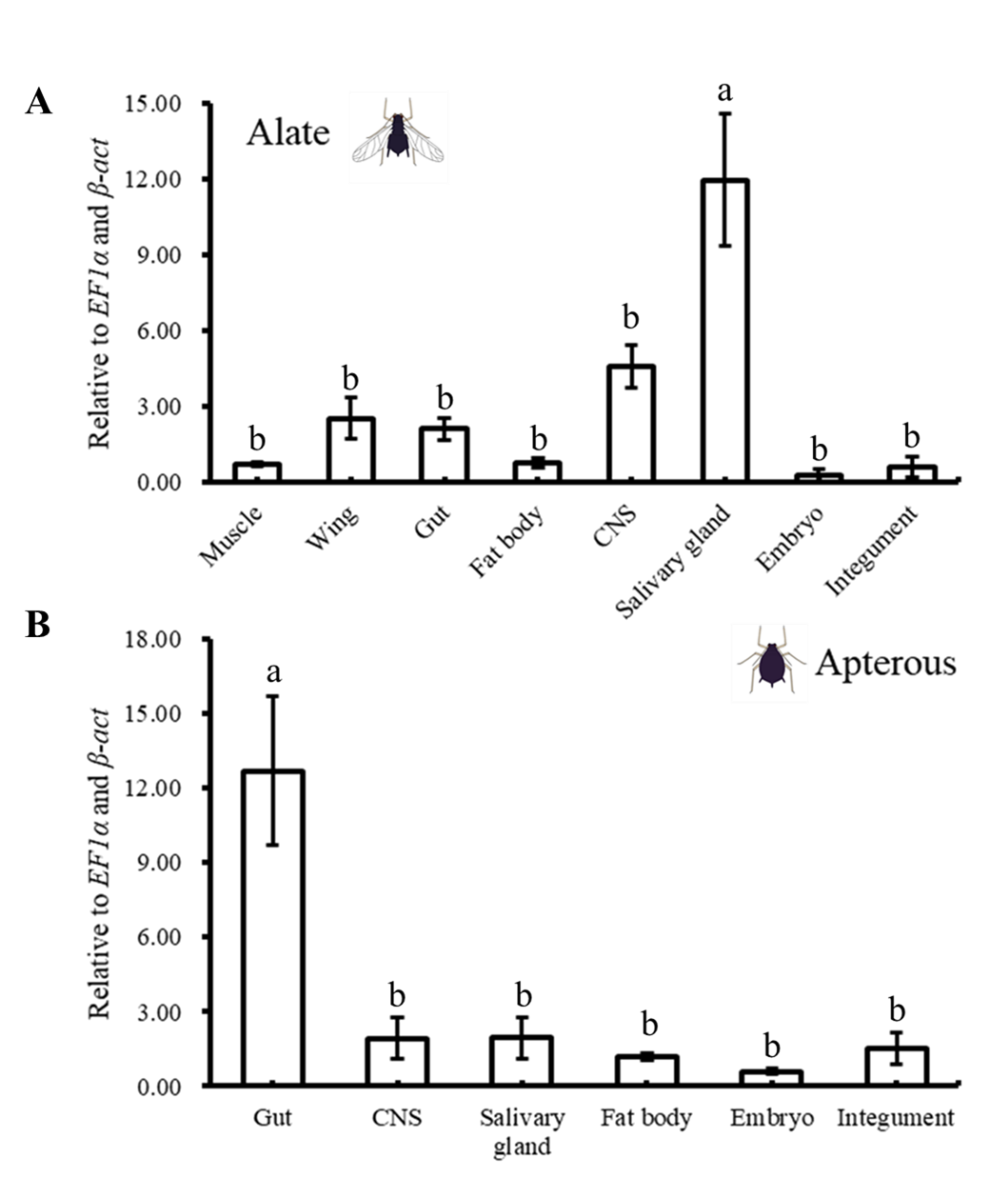
**

**Fig.S3.** Relative titer of AcMSV in different tissues of laboratory population of *Aphis citricidus*. (A) Viral titer of AcMSV in Muscle, Wing, CNS, Embryo, Fat body, Integument, Gut, Salivary gland of alate (wing morph). (B) Viral titer of AcMSV in CNS, Embryo, Fat body, Integument, Gut, Salivary gland of apterous (wingless morph). Different tissues of aphids are dissected and collected. Gut, muscle, fat body and wing of every 100 aphids, integument and embryo of every 50 aphids, CNS and salivary glands of every 200 aphids as one repeat. All tissues were dissected in frozen PBS at pH7.4, four replicates of samples of each group were performed. Data are means ± SE for four biological replications. Reference genes, *EF1α* and *β-act*, were used to normalize the expression of AcMSV by qBASE. Different letters above the bars indicate significant differences of AcMSV in tissues (Tukey, ANOVA, p < 0.05).

**Table S1.** A list of all primers used in this study

| **Primer name** | **Sequence(5’-3’)** | **F/R** | **Product size (bp)** | **Function** |
| --- | --- | --- | --- | --- |
| F1 | AGACTAACATTCACACAAACAACCAA | F | 2690bp | Sequence validation |
| R1 | CACTACCCAAATCTTCAATACTCTCA | R |  |  |
| F2 | TTTCTGAGAGTATTGAAGATTTGGGT | F | 1920bp |  |
| R2 | GGAAGGATTCAGGTTTCTTGTATGGT | R |  |  |
| F3 | ACGAAAAATGTAATAGCGATGAAGTT | F | 2427bp |  |
| R3 | CGACCTTACTTTCAATAGATAATAATACA | R |  |  |
| F4 | AGGAGGGTGTAACCGAAACTAATAAA | F | 2084bp |  |
| R4 | TATCGGCAAAAGTTATTGGTGTTATT | R |  |  |
| F5 | ACACAGAAGGCTCCATAATACA | F | 2450bp |  |
| R5 | TTACCAGAAGTTACACCACCAG | R |  |  |
| F6 | AACTCTAATAAATCTGAATGTGGACG | F | 2564bp |  |
| R6 | CGTCTATTGTCATCTACTGTGGAAAG | R |  |  |
| F7 | ACCTATCACTCCAACATCAGACTTTC | F | 2393bp |  |
| R7 | GACCGACACTTACATTAGAGAACCA | R |  |  |
| F8 | TACTGTAACCGCCTGTAGAAAGAAT | F | 3421 bp |  |
| R8 | CAGCCACAAGTGAAACTAGAATCCC | R |  |  |
| F9 | TGTTGAAGGCATACAAATGTTACGA | R | 2737bp |  |
| R9 | GCTCAAGATACAACTATTGTTACGGCTA | R |  |  |
| AcMSV-F | CCCTAACCTCGCTCGTACTG | F | 1101bp | Validation the presence of virus |
| AcMSV-R | GGTGTACCAGGGTGAAAACC | R |  |  |

F: forward

R: reverse

**Table S2.** Percentage amino acid identity of the replicase protein of Aphis citricidus meson-like virus with that of other mesoniviruses.

| **virus** | **Gene** | | | | |
| --- | --- | --- | --- | --- | --- |
|  | **ORF1a** | **ORF1b** | **3CL pro** | **Zm** | **HEL1** |
| KSaV | 11.3 | *18.6 | 20.1 | 14.4 | 39.8 |
| NDiV | 18.6 | 11.3 | 19.8 | 14.4 | 43.0 |
| DKNV | 16.2 | 16.2 | 20.4 | 14.4 | 43.0 |
| CASV | 17.6 | 17.6 | 19.8 | *17.8 | 33.6 |
| HanaV | *18.3 | 18.3 | 20.4 | 11.1 | *43.4 |
| OFAV | 17.4 | 17.4 | 19.5 | 10.0 | 35.8 |
| KADV | 17.0 | 17.0 | 19.5 | 14.4 | 33.1 |
| NseV | 17.3 | 17.3 | 19.5 | 17.8 | 32.2 |
| MenoV | 17.1 | 17.1 | *21.7 | 15.6 | 41.6 |

* Numbers in red represent highest identities of Aphis citricidus meson-like virus with other viruses

** The accession numbers of viruses are shown in Table S4

3CL pro: 3c-like protease

Zm: Zinc cluster-binding domain fused to HEL1

HEL: superfamily 1 helicase

**Table S3.** Pairwise evolutionary distances (PED) using MEGA 6.06 software based on conserved domains in ORF1b.

|  | **KSaV** | **NDiV** | **DKNV** | **CASV** | **HanaV** | **OFAV** | **KADV** | **NseV** | **MenoV** | **ACMV** |
| --- | --- | --- | --- | --- | --- | --- | --- | --- | --- | --- |
| **KSaV** |  |  |  |  |  |  |  |  |  |  |
| **NDiV** | 0.106 |  |  |  |  |  |  |  |  |  |
| **DKNV** | 0.073 | 0.105 |  |  |  |  |  |  |  |  |
| **CASV** | 0.151 | 0.127 | 0.151 |  |  |  |  |  |  |  |
| **HanaV** | 0.151 | 0.133 | 0.154 | 0.168 |  |  |  |  |  |  |
| **OFAV** | 0.342 | 0.337 | 0.335 | 0.336 | 0.347 |  |  |  |  |  |
| **KADV** | 0.288 | 0.282 | 0.286 | 0.288 | 0.305 | 0.314 |  |  |  |  |
| **NseV** | 0.240 | 0.233 | 0.238 | 0.238 | 0.253 | 0.342 | 0.295 |  |  |  |
| **MenoV** | 0.276 | 0.273 | 0.279 | 0.272 | 0.298 | 0.322 | 0.267 | 0.294 |  |  |
| **ACMV** | 0.709 | 0.706 | 0.710 | 0.710 | 0.704 | 0.716 | 0.705 | 0.710 | 0.702 |  |

**Table S4.** Virus GenBank accession numbers and abbreviations used in this study.

| **Family** | **Genus** | **virus** | **Accession Number** | **Abbreviation** |
| --- | --- | --- | --- | --- |
| *Arteriviridae* | *Muarterivirus* | Muarterivirus afrigant | NC035127.1 |  |
| *Arteriviridae* | *Alphaarterivirus* | Alphaarterivirus equid | X53459.3 | EAV |
| *Arteriviridae* | *Lambdaarterivirus* | Lambdaarterivirus afriporav | KP026921.1 | APRAV |
| *Arteriviridae* | *Deltaarterivirus* | Deltaarterivirus hemfev | AF180391.2 | SHFV |
| *Arteriviridae* | *Epsilonarterivirus* | Epsilonarterivirus hemcep | KM677927.1 | SHEV |
| *Arteriviridae* | *Etaarterivirus* | Etaarterivirus ugarco 1 | KC787630.1 | KRCV-1 |
| *Arteriviridae* | *Iotaarterivirus* | Iotaarterivirus kibreg 1 | JX473849.1 | KRTGV |
| *Arteriviridae* | *Thetaarterivirus* | Thetaarterivirus mikelba 1 | KM110938.1 | MYBV-1 |
| *Arteriviridae* | *Zetaarterivirus* | Zetaarterivirus ugarco 1 | KC787630.1 | KRCV-1 |
| *Arteriviridae* | *Betaarterivirus* | Betaarterivirus suid 1 | M96262.2 | PRRSV-1 |
| *Arteriviridae* | *Gammaarterivirus* | Gammaarterivirus lacdeh | U15146.1 | LDV |
| *Arteriviridae* | *Kappaarterivirus* | Kappaarterivirus wobum | JN116253.3 | WPDV |
| *Coronaviridae* | *Alphaletovirus* | Microhyla letovirus 1 | GECV01031551.1 | MLeV |
| *Coronaviridae* | *Alphacoronavirus* | Alphacoronavirus 1 | AJ271965.2 | TGEV |
| *Coronaviridae* | *Betacoronavirus* | Murine coronavirus | AY700211.1 | MHV |
| *Coronaviridae* | *Deltacoronavirus* | Bulbul coronavirus HKU11 | FJ376619.2 | BuCoV_HKU11 |
| *Coronaviridae* | *Gammacoronavirus* | Avian coronavirus | M95169.1 | IBV |
| *Medioniviridae* | *Turrinivirus* | Turrinivirus 1 | KX883629.1 | TurrNV |
| *Medioniviridae* | *Alphamesonivirus* | Alphamesonivirus 1 | DQ458789.2 | NDiV |
| *Mononiviridae* | *Alphamononivirus* | Planidovirus 1 | BK010449 | PSCNV |
| *Euroniviridae* | *Charybnivirus* | Charybnivirus 1 | KX883628.1 | CharNV |
| *Roniviridae* | *Paguronivirus* | Paguronivirus 1 | KX883627.1 | PagRV |
| *Tobaniviridae* | *Okavirus* | Gill-associated virus | AF227196.1 | GAV |
| *Tobaniviridae* | *Bafinivirus* | White bream virus | DQ898157.1 | WBV |
| *Tobaniviridae* | *Oncotshavirus* | Chinook salmon nidovirus 1 | KJ681496.1 | CSV |
| *Tobaniviridae* | *Bostovirus* | Bovine nidovirus 1 | KM589359.1 | BoNV |
| *Tobaniviridae* | *Infratovirus* | Infratovirus 1 | KX883638.1 | INTOV |
| *Tobaniviridae* | *Pregotovirus* | Ball python nidovirus 1 | KJ541759.1 | BPNV |
| *Tobaniviridae* | *Sectovirus* | Sectovirus 1 | KX883637.1 | SECTOV |
| *Tobaniviridae* | *Tiruvirus* | Shingleback nidovirus 1 | KX184715.1 | ShNV |
| *Tobaniviridae* | *Torovirus* | Equine torovirus | X52374.1 | EToV |
| *Mesoniviridae* | *Alphacoronavirus* | Alphamesonivirus 2 | KC807171.1 | KSaV |
| *Mesoniviridae* | *Alphacoronavirus* | Alphamesonivirus 3 | AB753015.2 | DKNV |
| *Mesoniviridae* | *Alphacoronavirus* | Alphamesonivirus 4 | KJ125489.1 | CASV |
| *Mesoniviridae* | *Alphacoronavirus* | Alphamesonivirus 5 | JQ957872.1 | HanaV |
| *Mesoniviridae* | *Alphacoronavirus* | Alphamesonivirus 6 | KT966491.1 | OFAV |
| *Mesoniviridae* | *Alphacoronavirus* | Alphamesonivirus 7 | KT966495.2 | KADV |
| *Mesoniviridae* | *Alphacoronavirus* | Alphamesonivirus 8 | JQ957874.1 | NseV |
| *Mesoniviridae* | *Alphacoronavirus* | Alphamesonivirus 9 | JQ957873.1 | MenoV |

**Table S5.** A list of primers used in race amplification

| **Primer name** | **Sequence (5’-3’)** |
| --- | --- |
| 3-GSPs1 | TTGTTTGTTTCGGGTGGGCTAT |
| 5-GSPs1 | ATGTATCCGTGAAAACTTATTGCG |
| M4Tm | GTCACAGTCACGACTTTTTTTTTTTTTTTTTT |

S6. Sequence of Aphis citricidus meson-like virus genome

>Aphis citricidus meson-like virus

CCAAACCTTTCTAAACTTTTCGCAGTTATCTGCTTGCCTTTAGAGGTTTGCCCACTAGGGTTTGCAGCTTATGTCTGCTTGCTAATTTATTAGCTTTCTACAAATCTGCGTTGTTTTTAACTACTCTTACTATCCCTTAGTACTAGGTAGACTAACATTCACACAAACAACCAACTCAACAGATTCCAACCATCCACATTTGAGTTTAAAGTGTAAGCTTTATATATATATCATTCAGTCTCTGTCTGTATATATACGCAATAAGTTTTCACGGATACATCATGTATCAACCAAATATAAAACTAAAATTAACCATGTATAACATGTTTTATTTGGTTTTACTTGCCTATTTCCTATACGATTTAACCAACATCTATTACAAGCGCTCGTATGAGTTTAATAACTTGAATAGTTTGGGACGTTACGTTTGGAAAACTGGCATATTGCACAAATTTATATTCATTTCCGACATCTTATATTGTGCTCTTAAACTTGGCTTAATTTTGACTGCAATACGTATACTTTTCATATTACAACAAGCTTTTTACAACTATTGCACTAAATTCGCTAAGTATAATGGACCTATTTTACCATCAGGTCTTTTTATTAAGCATTACGCTCGTATTCTGAATTTAATTTTCAAATTTTGTACCGGTTCTAACCTTTTCTATTACGCAGACATGTTAGATACAAAACATTCTAGATCCTACGTGATAACTAGAAATAATATTCTCTACAATGGTATGACGAAAATCAACGGTTTTTGTTCTACATGTTATGCTTTCGGTCATTATCAACATCAGCACGAACGTACATATTTAAGCGATATGTCCATTTTTATCGCTAAACAGTGTCGTATATTTAGTTTCAATCAACTTGAACAACGTTACAATCCAGTTCTCGCCGCACCAACTGACGTTCCACAACTCTTTACATTCGAGCGCGTTTTGGATTTCTTTAACCACATTGAAACCTATTTTATACCCACACAAAAGTCCAACCAAACCATTAATAAAAATCAATTAACTGTACACAATATTATATGGCCCGAACCTACAGCTTCAGCCATTGCTTTAACTCGTGTTATTAATGGAGTGGTTGGTGATGCTTTATACGAATCTACTAAATTCGGTATTACTTATGTAAATACTGATAATCGTATACCACACTATATATTGATTAAATACAACGATAACAATCTACATCAAACATTACAACGTTTGTCATTATATGGTGTTAAAGATGTTACTATTTATGGTAGTAAGATCGATAGTGTTGTTCTTGAAAATTATGGTTTTAGATCAGTAGGTGTAGCTCATGGTAACTTGGATGTTTTCCCTGTTGTCAATGATGTATTATACCCTATAGTGGTGCGTAATTTACGTACTATGTATAACTTTTCTGATATAACTTTAGCTGGTCTAAAATATGGTAGTAAATATAGTGATATTTTATTGGAAGAATCTACTACAGTAGACGAAACAGTATACTCATATACAAACGAACTTAACACTGAAGCTGAAAATACTAATAATGAACTGTTAGATTTGTCTGATAAAAGTGTTGTGGTTGATAACTCAATAATAATAGATAACAATCAAACCTCCACAGCCGCAGACGGTCCTATAACACGTTCTGATAGTTTAACCAGTTTGTCTACTGTTAACACTATTATATTCGATGACATTGAACTCATAACTATAGCTGAAAATTTAAATGCTCTATTCCCAAACAAAGATATACGTTTAACTTACCAAGAATTATGTAAAGCATATAAAAATAATTACAATAGCGATTTACCTAATATAAATGGTTTTCGCGGTAAATTTTTATTTAGCTGTGGCATTACTAAACGTAACAGTAATGGACACGAATATTACAAACGTAACAAATTTTATAACGCGGAAGAGCTTGTAGTTCAAGTACGAAAATTTACTAAAACTATAGATAACCATAACAACACTCGTCGAGTACGAAATACAAGTATATTTTCAAATTTAACTTTTACTATTGTCTTGATACTCGGTTTTATTTGCGGCGCTTTAAGCACTAATGAAAATCTTGCAACATATAAATGTGAGAACTTTAAACAAGATTTTTCAGCTCCATTGTGTAGTACTTTCGGTGTAAATTATGATATAATTAAACAACAAGATTCCTTGACATACCAACTCAACACACAAACTTTATTATTACTAAACCATGTAAATGATGTTAAGTTGGATACTATAGATATGTCTTCACGTTGTTTTTTCACTTCTGACACCGTCTACACACACATTGATGAATATTTTGAAGCCGCTTATAATGCTGAAATAGGAGGTGAACTTTCAACTTTAACAGAACTTTTCAGAGATAAAGATCATCTAAACTTTTATAATCCTGTAAACGATTACACACCTTATAAACCAGCTATTGTACAGGACCATAGTTATAAACACTATGTAGTAGAACTTTTCAACAGTAAAGAATCTTATAAAATCGCTTTTACCAATAATAACGATAGACAAATACTATACACACACCCTACATGCATAACCACTTGTTATTGCAACCAATACAGTTTTATATCCGGTGATGATGTTATAGGTTATGATTATAAGACAACTAATATCAATGACTTAGCTATGCGCTATATATCATATAAAGGAGACTTAAGTAGTAAAACTTATGAACTCTATGAAACATTTGAAACTAAACTTAACTCTTATAATCCTCTACAAGTTGGTGTAGAACATATAAACGATTTCGACAACATTAAGATTCTTACTAGTCCTGTAATACAACGCAACAATAAAGCTTTAAGCATTTCTGAGAGTATTGAAGATTTGGGTAGTGTTAACATCAACTATTTAACATATAAAACTACATCTGATCTTTGCACAGGTAAATATAATAAGCGTACCACATATTGTAGTGTAGAACGTGAAAACTTACCCTTAACTTTACATACTACATATGATGGTGATTATTTATTGGAGTGTTTAACTTTTGATGACACTTGCAATACATACGCTAAATTACTTGTAACTAGTTACGATAACGTAGCTTATGACGAATTTAAGAAAGCTCATCCGGACCTATATACATTACCTAAAGCTGAAACTAATGACTTAACTTTCGAAGATCTATATTACTTATACTTAGATGATTTTGGTAATGTATTAGTTGACACGCCTAAAGCTGAGTTGCAAGCTATGTTCTATTCTAAATATATAGAACCCATAAATAATATACAAGAACTCAAACTCGATCAAGTAAATCAACATTATATTAATTGTGAGTTTTTGTCTTTATTATCTGGTTATACTTGTGCTATTAAGAGAGATTCTAATTATTGTAACTATTATAGTGAATTGACTTCATTTAACCTATATTGTAAACAACTTACTATTGAATTTCATAATTTGTTACACTATATTAGTATAAATTTTGCTAAAGTATTTAATTATATACATGGAAAACAGTACGAATTTAACAAAATTTATGAAACATATACAACTAATGTCTTAAATAACAACCCTAACCTCGCTCGTACTGTGAGTAATATGGGCTTTACAAATGTTAGTATATGCTCTTTGTATGCACATCCAGATACTGAATATATCCACGACTTATGCCAAACTTACAATATGGAAAATTATAAAATATCCTATGTCAAACCCAGTATTTATAATATATTTTATGTTAAATACACTGAACATGTCGAAGAGTTAACTATAAACAGTTATTATCAAATCAAAGACTCTATAGCTTATAAATTGCTTGATAAATACGACAATGTTGTTGTTGAGAAATTTTTAGCTAGACAATTATTCTACGAACCTAATACCTATACCCTAGCATATTCTCATGTAGACAACCGTCTACAATATACTTGGGGTTTACGTAATGATTACCAAAACTTTATATTAGAAGACACAAACAAAGTCATAGCTTATACAGGTTTTCATTTTATAGACATCGGTATTATCGGTTATTTAAATTTTAATAATGTAGGTATTGTTATTAGCATTAACATCGTATTTTATCTATTAACACGTAATAACAATCCTAGTTTTGACATAGCAACTTGCCTACTCACCTTTATTGTATGTTGTTTTAAAGATACTTATTTGGCTATAAGTTTATTCAATCTTAACTTGAATTTTATCTTACAATTGTATGGTATAGCAACTATAATTTATACAACTATACGTCTACATGACTATTATTTGGTACGTACTACACGCTTAGGTCGCGATTATTTCGTTATGTTAGCACAGATTACAACAAATTTATTAACTATCTTTAACATATTATGGGGCTCAACTTTACTAAACATGACTTTCGTATTAATAGCTATTGTTTTGTTGTATAGACATTTGTCTAATACTACTAACAAGAAATACTGGTCACTACCTTACATCAAAGCACCATTAGCTTCGAAAATTTGGAACAAATATCGTGATAACGAAAAATGTAATAGCGATGAAGTTTATTTTAATAAAATAAGCCGTGAATTTAAATATAATATACAGAATAATAACTTAAATGAACGTACAAAAGACCTATATTTGCTTCAGGAGATATACCGTCATATAATTATTGGCAATTCTAAGGGTTTTCACCCTGGTACACCTAATTTGGTTGTAGCTAATTATAATGTGGCTAGTGAATCAACTATTAACTTAAAACCTGAAGCCTTAACATCTAATTGCATACCATACAAGAAACCTGAATCCTTCCAAAGCAATCACGTATTCGAGGTTAGTTACAGCGGTGCCAATGACGGTTTAACACGTTCTTTGGGTGCTATAGTTGTAAACAATAACTTATATATATTACGTCATTTATTGGGTGAAACACATATTGACTTTAACAACCCAGATTGGAATTGTATAAAAGCTACTAAAGATAATGACGTTTTGAAAGATTTTAACTTTGCTGCAGCGCACTATGATGGTAAACAGTTTTGGATTGTACCAGTCAATACACATGCGCACGAAAAATACAAAACTTCAATTACAATTTACGAACAACGCGTACGTAGAGCCCCTATGTACACGGGTTATGCTGCTTTGTGGGATTTTAAACTACAAAGCTGGATATCAGGTTATGCATCTTGCGGAGCACATGATATTTCTACTTTACCTGGCTTATGTGGTGCACCATTATTTTCTAATAGTGGCCACTTAATAGGTATACATATAGCATCATCTACACAGAAAATTGATAGCACTAATCCTTGGTACGACCTTATCGGAGATACTATAACTACAAACTATTACACAGATATATTAGGAAACGTACCTAAAGATATTATAGGTTACGATTGTAATAGTGCTTGCCCAATAGGTTTACAACATCCAGTTTTGAATCCATTTGGCGCTTTAGGTGCTTTAATTGGATTTACTTCTGATAAACCCAACGCTAACAAAAATTACGACCAACGTTTTTACACATATTTTGGTATTGACCAAGATATGCATTCTAACTACACCAGTAATGGTATTAACTTTGATTTGGAATATTTTCTAGCTAACAGTAAAGATGTTATGCTTAAAACGTTTGGTGCTGGTCACTTAACATTTTTGGGTTTATTACAAATAGAAGGTTATAAATCTGGTCAGGGCAATTACGATCTAAAACAATGCTATAATAATTATGAGAAATACCGTAACAAACTAAAACCTGAAAGTGCTATCGCTTGTTTTGAGGATAAAACAATAAACGGAAATAACCGTTATTTTTATCTTTTCAGTGTAATGGAGATTGTACATATTATATTAAAAGTTTTTAAACTTGATTATACAATTGTTAACTTTATCTTTACAATCTCTTTACATTGTTTAACTGTTATGCAAATTATAACTATTATTTTAACTTTGTATAAATTAATAACTGAGAATAATAGTATTTTTAATAAAATTTTAAAATTCATTTTGAATTTTATAATGTTAGTACTATTGTTTTTAGATAATTATCATGTTTTTATGCGTCTTATAGTCAATAGTTCTGACTTTACCAACATATTTAGCTCCTGGCTGTATAAAACTGATATATCTGAATACGGTATAATAACATGCTATAACAGAAATTGGTCCTTACTTGATAATAATAAAGCTTTAAATATATTTAATAAAGGAGCTGAATTTTCTTGTAAACTTAACTTTTGTATGGTTAATACTAAAAAACCTAAAACATTCACCAAGAAACTTATAATGAACATCTTTGGTTTACCAGCTTTTGATTATCTTTGTATTATGGACATTAAAACTAAAGCCACGTATAGCTATTTATATGTAACACTGTATGAATACTTACCAACTTATATTATAACTTACATACAAGTATTAATAAATTATTATTTTACTGTCGTACTATTATTACAATATTCAACCTTTTTAAACAAGCGTTTTAATAAATTTAAATTATCTTTGACTTGTTTTATTAAGCGCTTAATTTCCAAATATACTGTTAGAGTAAAACCAAATGCCATTTTGGTCAATTTGAAGCAGGAGGGTGTAACCGAAACTAATAAAATATCAACTATATTGAACGCCCTAACCGCACTCGTCAAATTCGAGCAGTATAAACATCTAAAACCTTTACAGGTTAAACTTTTAAGTATTTTGGAAATCAGTAAAGACGAATTAGAATTTGAATTTGTTCTTAATATATTACGCGAAGAATTTCATGATTTATACAAAATTATACATCCCCTTTTGGTTAAGGGTGTCTCGCATGAAGTCATTTCTAGCGTAATACGTGATGGTTACATAACAACCGGTGCTTATGATTTAGCTTATATTGAAAACAACTTAACTAAGCTTATGACTACTCTAAGTTCTCTTGATAACGATAAAGATGTATTATTATCTATTGAAAGTAAGGTCGAATTCGACTTACATTATAATAATATAAAGAGTAATCTAAATTCTATTACACGTGATATTTTCGACTATAATATTGATGAACTTATTAATTCCATAGCCTCATGTGAAGGTAATGAAATAACCGACGCATTTTTAGAGATTAGTACTCTCATTGAGGAAGTTGATAGTCTGCAAAAATCCAAAGATAACAATTATTTCAAAGGTAGACTTAATAAAGCTTGTTCCACATTACGCAAGCTTAATAATATATATATGGAAGAATTGAAACGTGAAGAATTACAAGCTATCAAACTTAATAAGAAAGAACAAAATCGATTAGCTGATGAGCGAGCACGTGAATTAACACAACGTAATAAAATAACTAATATAACTCGTGCTATGCTTATTATATTAAATACTATACGAGTTGCTAACTTAACTGATAATAAAGAATTAATTCTTGATAAATTGGATAACACTAAGCGTGAAGCTTTAATTAAATTATACGAAGAACTTAACAAAGATAAATATCAACAGCGTAGTGAATTAATTACAGCCGAAAACTTTTTAGATATACCTGATATAAACGTTAGCTATTTTGCACCACATCGTTCAACTATGAGTGTTATTGCTGAAGCTAAAGGTATATTTTTATGGAATGATACCTATTGTGAAGAAACTTTAACTATCTGTGGTGAAACACTAATTTGTACCTTACCTCATAAACATTCCATAACCAATTGTTATAAAACACATATGCATCTTTATTATGAACATTTAAATGAATGTAAGTTATGTTTTAAAAAGTTTATACAACGTAAACATCCAAGATGCGGTGCTATTTACACTAATGAAGAAATAAGAACTAATCCTGGATACTTATTCATCAACCATTTAACCCGTTGGCGTAGCTGTAAAGCTTGTATTACTTGCAGTTATTGTCCTAAAGGAACCAAGCAAGCAAATTGTGACACAGGTTCTTGGCATACTAAATCCATTGATGTTACTAACTTATTTATGAAACCTATACCTGAAACCTGGATTAGTAGTAATGTTGATAAAGGTGAAATGACTATTAATATTGATAAAACAGGAGTTCTTAAAATTAGCGATAAAAGTTGTAAAGTCGATTATGTAGTGGCAGTACCTAAAAATTTAAATCTAATTATAAATAACATGACCACCCATAACCGTCTGACTCATGCTAATTATAATATATACTTCAAAAAGAATATAAGCGACAGTGCTTTATTGAATGCTTATTATTACCGCATGAAAAACTTAATTGAAGAACAAACTATTTATAATAATGCGGTTAATGATATTCTTAAAGCTGAATCTAGTATTTTACCAATACCAGTCGCAGATGATGTAAAACCAAAACAAATTTTTCTTCATTAATAGTTGATGATGAGGCTATCAACTATTCTTATCCTCTTTTAAGGCCTCACCAACTAAAAATCAAAGACCATAACAACAAAATTCATTATACTCTGGACTACTCGATTAACAAAACTTCTAAAACATATATCTATAACGGATTTTTAATCAATTACAAATATAAGAGTACCACAGCATATGAATATGAAGTCTATTTACATATATTGAAACTTCTAGAAAATAGCGCTGACAAATTCATTTTACGTCACTATGCTATAATGGACACAGAAGGCTCCATAATACAACTTGTTCGTTATAAAATAACACCAATAACTTTTGCCGATATATATGCCATCATTAAAACTGGCGATTGGACTCTTATAAGTAAGGGTTTAGAGTACGTCACATGGTTTAAATTTAAATACACATATCAACACAACCCTCCCAGACACAGTTGTTGTCTGCAATGTCGAAGATATTTATCCGAAATGGGTTTATTACTTCATACTATTAACCAAAAACTTAGAGCTACTGTCAAGAAACTTTTACGACATTATAACTTCCGAGTGACAGCAGATAACGTAGATTTAAATGGTTTAATAGATTTTGAGGATTTTACTAAAATTAAACGACGTACTTTAGGTGAGATTGATAATATTATTGAAGATGTTATGGCACCTTTTTCACATATGTTTTATTCATATTATGAACAAACTGGTTGTTACTTCATATCCTCACCTATATACCCTATTAATACAATCCTAACTTTAGATAATTACCGTACAGCGGTTTATAATAACTCCAACGATTGTATATTTAGACCAAGTTATGAATCTTTTGTTGAATTTTTAAACCTTAAACAATATTTTAACATACAACCTAAACAAGATATCTATAACTTTTGGGCTAACATTAAAACACGTGAATATCCAAAGGGTTTAAATTACCATATACACCCTATTATAAACGAACGCACATACATTGATTTGTCTAGTTTTTATAATTTTGATATTGAGTCTTTAACCAATTCTGAATTATATATAAAAACTACATATAAAGAATCATTAATACAAACTAATCTCGATCTTTTGTACAGTTTATCCGGAAACAATACTATATACTTATATGTCTACGATCACCCTGATATTTCTAACTTTCACAAACGTAGTCTAGAGTTAGTTTCCAATTATTGGATAAACCATTTATATGATGCAAACGTCAATTTATCTCATTTTAATGATATACTTAATTATTCCAATACAGGGTTTGTGAAATACCCTATTATAGGTTCTTTACCAGCCAAACCTTTAGCTAAGTGTGATGAGTGTAGTGTTGATAAAGACATTAGTGAAATTTATGATCATGGAACTTTGGACAATGCCTTACACTTTTTAGATCCTGAAACTGTAAACTTTAAATCTGTTAATCCTTTAACAGAATATGATCAAGCACTCATGTATATAGGTGATTTTTATGAAAATTCACCTTTTACACACGAACCTAATTTAAACCCTGGTTTAATTATTAATTGGAATTTATTGGATTACTTCAAACGACATGGTCTTACTATACATATACCTCCAGCACAAGCGGAACCTTTGGACGATGCTGAAACAACTTATCAATCAAGTTATTATTTCAGACCACCAACCTATAACGGAGTCGTGGATGATTTAAATTTGTTTAACTTAAATTCAGCTGGGTCTATTTCACCCGTAAATTTATTAATGTGCTATGAATACGTTCTACATAAATTAAGATCGCGTGTCGTTGCAACTGATGGTAAACCTAGTATAGTTTTACCTACTTCAACTATAAAAGTCCGTAATCCACACAAATCATCCGGCATACCCTATCGTAATTATGGTGATGCCGAGTTTATGCGCGATTTATATGGTAAAGAGAGAGACAAAATAACATTACATAAAACCCATTCAGCCGATCCATCATTTACTTTGGTTATTAATAAAGTAGCTATATCTACTAAACCAAGAGATCGAACTATATTGGCTATTAACTCTAATAAATCTGAATGTGGACGTCGTTTATATCGCAACTTATTAGAAAAGATTAAATACTCCGCGAAGCGTGGTGGTCCCATACTTATAGGTTTTAGTCCTATGTATAATGGCTGGGATAATTTCTTCAAACAATTAGACAACGCTTTTAATAATAATAAATATACATTACGTGGAGGTAAGGATTACCCTAAATGGGATCGTAAAGTTTCTAACTTAATACAATTTGTAGCCTCATCTATCTTCTTTATGTTACAAGACCCATACAGTGTTAATGAACATTGTAATGGTGAAAGTTTACATGATTTATTTAATGAATTTTTAGCTGAAACTTCTCAAATAATATACGATTTCCTTATTTATGACAAATCATTATATCAAAAACCTGGTGGTGTAACTTCTGGTAATTCACGCACAGCTGATGGTAATTCATTTTGCCATCTTATATTTGAGGCTAATGCGACACTTATGCAACTATCTAAATCTACCAGTGAAAATTTTGATTTATACCGCGATATACGTGATGAGATTTCATATTACATGTTTAACACTCCAGCTCATTACTTAAATTGTCAACCTTATTTCAACAACCCTAAAACTATAGATTATATAGAAACACACATATCACGTATCTTGGTCCTTAGTGACGATGCTGTGTGCAATTTTGATTCACGTGTTATAGATTATGACGACCTTATGGCTGAATCCCTAATGATATCTAATTACGATATGCCTATGAACAAAGAAAAATACCATGTAGTTCCTATACATGAAGGTGCTAAAGATTTCTTGTCACAAGAAACTTTTAATTATAAAGGGAAATTTTATCCATTACCTAACTTCGAACGTGTTGTTGGTGCTTTAGTTTTAGATACCAGTACTAACACGTATAACCCTAAAATTGAACTAGCACGTACTTTCGCATTATACTCTTGTTTATATCCTTATCTAAAGATTGAGGGACATAAAAAGGAAAAACAGTTTATCAAAACTTTGGGTCGATATATAGACGAACATCCGTATAATATAGACATAGACACTGTTAATCAACTTAACTTATTTGATTACCTTGAAATTGATACTAGCGTAGATTTGAAATCTAACCGTGATTTATTTTTGGATAGATTATACGGTTATGATGTAGCTGACGAAGAATATAATTTAACAGCCGAATCTGTAAAACTTAGCAATTGTTTCTTATGCGGTCAGCAATCCATGCTTGTCTGCCTTACTTGTCGTTTGACATACTGTAACAACTTAACTAATTCTCATCTTATAACGCATATGAAGTTGACTAAACATTACGAGTATGCGACAGTTTCAGGTAGACGTATTCGTTGTAACAAATGTAACGAATCCGATATTAACCATTTATATCACAGTACTAATGATACAATAACATGTCTAAAACATAACATAAAACCTAATCCAAAATCACTTATATGCGATGATCGTTTATTACTATACAGTGATAATAAGTCTAATTGTGTCGGTCAACTTAACTACGATTTATTATATGCTTTTTATAAAGCTTATGCTGTTAACAATATTACAGAAACTATAAAAATATTAATAACTTTATCAGTTAATGGCACACCTTATCCTTACTATCGATACGTACGTGACTTAACACGTATAGAATATAAAAAGTTGAAAACTGAAGAAAATATAATCTATATACAAGTTAAAAATTTTGATTCTTTTAATAATAAAGTAACTGTTACACTACCACCAAATACACGTATAAACCAACATCATGAATATAATCTTATATATAACCTGAATGATGCTGATGGTAACTACAAATATATACCTATCACTCCAACATCAGACTTTCTAATAGATGGTACTAAGTACTATACTATTTGGTATTTTAATGTACCTGAAGGTTTAATAGACATTTATAAGTGTAAACAGATTGTATCTAAGCCTATAGATACTCTTGGCGATGCTATAGATAGAGGTCGTACTGACTTACCTACTATTTTTACTAAATTCTTCAGTTTTGAAAATAATCAGCCAAATTTAATATTCAATTACCAATCTACTGGTAATTATTATAACATGGATTTACTTGTGAAATTAGTACGCACTAATCAATTTAACATAGTACAGGGTCCACCTGGTTGTGGTAAAACTTATCTAGCTTCGCATTTTGTAAAACTTATGATTAACAACTCCAAACGTGTATTATTTTATGCACCTTCTCATAAAGCTGTTAATGTTATGTTAAATAAGTGCTGCGCATTATTTCCAAGTACTCAACATAGTAAACTATTTAACCGTGTTATAAGTAATGACAAAAACGAAAACGTACAACGTGATATACCCAATTCAGTACCTATACGTCAATCTGCAACTTATCTCGAATTGGTCACATTTTGTACAGTACAAAGCTTCAAGGCCTGCCAACATATACAACCAGACGTTGTAATAATAGATGAATTTTCACAACTATCAGATTTTTATTTATTTTTGCTTATGCAAAATTTACCTAGCAAAACATCTATTATATATTTTGGCGACCAATATCAACTTTCCACAGTAGATGACAATAGACGTAATTTACCGGTTGATTATAAAAATCTCATTAATTATAATGCTTGTAAATACAACCGTTTAAAAAGTGATAATAATCCTTATTTATTATTGAAGGATCATTACAGATGTCACCCAGATATATGCGATCTGGTTTCTACATATACATATAATAAAACTTTAAATTGTAAAGTTGATAAATCAGAACGCGAAACCATAACTAATATAGTTAGTACTAGCGCTATACATATTTATTTTAGTACTTTAACTGATGAAGAAAGACGTTCTAGCGTAGCAGGTGTATATTACAACGAAAGTGAATATAAACAAACAATACAACTTATAAATAATAATGTCGTACCACATCTACGTAGTACTGTGGCTATACTTTGTTGTTATAATTCACAGTGCGAACGTTTTATAGTCGCACAAAGGAACAACTTAATTCCAGCTAATATACGCATTTGTACTATAGATTCTTCACAAGGTGATGAGTTTGATTATGTTTATTTATGTTTTACGCGTGTTAACAATTTCACTTTAGACCCTTGTCGTCTAAATGTAGCTATATCTAGAGCACGTTGTGACTTGTATTTGACATTACCTAGTGAAGGCTATAAATTATTACCAACTAATTTCCATAATCCTAAATATTTGCTCGATGTTAAACTTATTTTAAACAGCGAAGCTAATTTAAAAGATTTACATTTAAACAATTTACGTGATGAGCAATTATTTAATAAGCAACCAGCTAAATTATTAAGTCCTATATATAGCGATTACTTCGTTTTGGACGTCGAATTTGTTAACTTTTACAATAGCGTTGCTAAAAATTATTCCATACCTTTACAAACTAGTTTGCGTAACAATGTTGTCTCACAAAATTTATGTGGACAACCTATTATTTATCATGACAACCTTAAACCCTTTGTATTAGATATGAAGGATAACTTAAAATTTATAGATAAATATCCTAAACACATGAAACAGGAACTTATATTATCCAGAAAAGCTTTATTTCGTGATGTACAAAAAGCTAGTTCTTTAGGTAACCAAGTAGATTTTCTATACATAGTTCGTTTTATACATAAACATACTACTTGTGTTCCCGTTTTAGTTACTTGGAGTGGTGATAAAGATTATCCATTTTTCCACCCTTATACTATTTATGAAAGTTTAAAATGTAGCGTGTGCAAATATCCAGCATCTTTTGCTACGAAAGAACGTCAAGCTTTTTGTACTTACCATTCAAAAACTATTAACAATATACATTATTTGGTCAATCCACGTCTAATAGATATAAACTTATTTTGTGACCAGACTGGTATGTGTAAATTTTTAATTCGTAACACACTTAACATACCTAAGTCTTCTTCAGATAGTTTTGATATTGGTAATAAACAACTTTATCCTGAGCGTAACAATACATACTATAACTTAACTATGGCTCACTCATTAATTTGTACAGAAAATCACGGTTCAGCACATGATGCTACCGTAGATACTAATATGACTTATTGTCTTTTTCAATATTTATGGTATAATGAATACAATTATTTCGAACAACTTTTCGATAATACTATGCGTTTTCGTGATTATGATCCTACTGTAACCGCCTGTAGAAAGAATTTTCTTAATTCTTGGTTCTCTAATGTAAGTGTCGGTCACTTTTGTGAGCTTGGCGGTGGTAAACACCCTCGTCCAGGTTATACGCATAACGTTGACCAGATCAGGTTTGTAGATAATATTCAGGAGGATATGAATTTACATGTGTGTGATATTCCTCTTGAAGTCTACACAGATGCTTATTATTACCGAACCAAACACACTAGTAAGGAAGCTCTTATATTTTCGGATTGCAATTTGGACCACTATGATATACATACAACGAAGTATAAAACTAACTATATATACAAATACTCACGAACATATAAATATTCTACGAATAGTGGTTTGTTTTTAGATAAAGCTGTTATTAACGGACCTAATTTATTTTATGATTTTATACCTTATACAAAGTGTTCCGGTAAACATGTTTTTATTAACGAAATTCTGGATAATTTGGTTATACCCTGTAAAATACCAGCTCTTGTTGGTTCGACTATTTGTACTGAACATTACAAACATTACGATAAATTTAAACAAATAGCTATATTAACAAAATTTGGTTTCAGGTTTACACATTTTAATAAACAAACTATCAAGCCTGAAGCTAATCCTTTTACTATCGTACCAACTACTTTTAACCCTAAAATTGATAAATTTATACCTGGTTATGAAAATCGTGGTAAAATATCTAGCCGATCAAAATCTACTCAAAAGGCTATTCAAATCTTAAATAACTATATACCTTTACAAAACATTTTAAATTTAGGTTTGAATTACCCTAAGAATTTAGATTATGTATTTTTCGGTGCGGCTGGTTATACAGGTATTACACCTATGGCTAATGTTTTTAAAACTATTTTTAAATATAACTTAAATTTGGTAGACCCACGCTTTCAACATTTCAAAACCTGTAATGACATTAACTTAAAATACCACGCCAACGAAATAAAAACTTATAACTCTTCTAATATGGTATATTTAATTATATCTGATGTCTATAATTCCACAGATATAACTTGGTTTAATGATTTGATATATTTTACAAACTATAACTTATACGAAGATGGTTCACTTATATTCAAGATAACATCCTGTTTCGATTCTTGGGATCTTCTTAATAAATTATCACAATCGTTTAAAATAGCGAAGATAGAACGTTTACCTATTACAGGTCTATCTTCTGAATTGTGGGTTTTTATGTTGGGTTTTACCTCTAAAACTCCTAATGGTCCTAATAATAATTTTAAAACATTGGCTTATAATATTTGGTACTCTATGTACTTGGGTGTGGGATTAGATTCTACAGCAGAATCATTGAAATACAAATCTATTGTATTTTCAGACTATCCCTTAGTACTAGATAGATGAACATTCACTTCAAAACATTTATAATATTTTCCTTATTAGTTCAATTTTCATTTACAACTAAAACAGCTAATAAATTCTTACATTTATACACCGATTCACATAATAACATCGGATTAAATTTAAGTAAATCACTTAATAAAACTATATACATCTATTCTACTTCACCAATTTGGAAAGTAAATACAACTTCTGATTACAACTGCTATAAAATAACATACACTTATTTCAATTCTTCTGTTTATTACGGTTATTTTGATTATTTGTTACAATCTGTCATATACTCATATACACAATATAACCAGTATTCTACTTATAAATTTCAAAACGAAGATTTAGGTTTTGCGTATCATGTCTGATGGTAAAATTATAAATAAACGTATTAACACAACTAACAATAAGAATACTAAGAATAACAATAACAGGAATAATCGAAATAAGATTAACAATAACAATCCTAGTCAAAGAGTGTATCAGAATACTAATAAATATTGGGTGCAGCCACAACCACCACACCCAAACGCTGTGTATTTACCTTATCCACAAGTACCACAATATATAGTACAACAACCTAACAGCAATAAAAATAATAAAGTTTTTCAAAAACAACGTAATAGTCGTAGTAAAAGTTCTACGCGTGCACAAAGCAGAAGTAACTCCCAAACTAAATCTATACAACGTACACGTAGTCAATCTAGACAACGTACAACTTCTGTTAACTTTAATATCAATACAACTAAAAGCACTGATAATAACGCGTCACATAATATCGGACCAGAGCAAGATGCCATTAAATATCTACGTTGGCGTAAAAGCACTAATCATAATGGCCGTCCTGTTACATCTGCGAAGTTCCCTTTCGCCTTACCTATGGCGAATAGGATTGACCAATGTCTTGTACGAAGCAAACTTAATGACGATATTTTTGCTCTCTGTTTTAATTTGCAACATTCCGCTTTCTACAATGACTCATTATGGTCTAAAACTAACCATAAACCTAGTGCAGATGATGCTCAGCATCTTCTCTCAATTGTACGTACAGCTTGTGACGTCTATATTGATCGCCTTAAAATACTCTTATCTTCTGATAATACTGGCGGCACTAATAGTGTTATTACCATCAGCCAAGGCTGAATTTGATTTGAAAAACTTATTATATCATAAAGCTATAAATAAAGCTTATTTCTATGATAATCACGTAAACATAACTATTACTACACATTTTAATCGTGATTTCGTAGATAGTTTAAACCCATTAGTTAAAAATATATATGATATACATTTACAAGCTGTTGAAAAATCTTGTGAAACAACTTATACCAAACTTTTAATAGAAAGCGTTAATAATGCATTGGATCGTATATCTGTCAGACGTATTAAACCCTCTGAGGTTTTTGCTTTTGATTGTATTAAATTTGATTTATCTTCTTACGCAGCTTGCTTTTACGGTCCCAATGTTAAACGCATTGATTATGTCTTTCCACGTTTAGAAACAGTCGCTTTATGTAGAGATTTATTCTCAACTGTAACTTTTCTTGATTATGAATTATTAATTAACAATACCTTAGCTGGCTTACCTTACGATCATACCAATTTACAATGTCTGCGTGATTATTATCAAAATATATTTAATACACCAGCTAATTTAAGAACTATATTAAACAACGATTTGAAACTTTGTGTACAAAGCAGTAATAACATTGACCATGGTTCGGACCGTTCTAAACGTGAAATAAATCCCTATAACGACTTACATTATTTTGACGATTACGAAGATCAGCGTAGTGATTTGTTGAAGGCATACAAATGTTACGATTATTTACCACCTGGACCAGCCCTTACGGCTACAGAATTTCGTGAAACTGACGCCTACAAATATTCATCTGTGGGACGCCTATATATTGACAACCTACTGGCGTGTACCGAATTGGGTTATTTTAATACTAGTATATTATATGTCGATTTAAACGACATTTCTAATCGGTACCCGTTATCCCAATTCGACAAGAAAAGTCGCAATAAGCGCTGGGATTCTAGTTTCACTTGTGGCTGGCCTTTAGTGTCTTCTTTCACTAAATTAGTCGGTGGTGAGTGTGAAATGACAACTGACGTTAGTGCTGTAAAAACCAGCCTAAACACACTCAATACAATGGTAAATCAACAGAATGTGGTTTTGGATAACTTTCATAAATCTTTTGTAATCGAACATAAACAAGTTTATAAATTAGCTAATGAGCTTAGTTTAATAAACAAAAATCTTAGACAACTTACAACTTTAACCAAATCCTTTATTGTTGAATCTAATGACGCTATGGCTAATATAACCCAAAATATCATTTGTAATTCAATAAATATAAATTCAAATTATTTAAACAGCAAACTTTTACAAATTGTTGATACTTTTGATAGCAATTATAACCGATTCTTAGCTATGTTTACTATTGATAAAAACAATGATAATTTACCCAACTTTTCTTTAACTTCTTATGCAAGTAGTCAATTACTTAAGTATGGTGTTTCTACTGATTTCCGTCATGCTAAATTTATAGTTTTAAATACGGAACGCACCTTAATTGATAACTTTTCTGAAATAAAATTTAATGGGTTATTACCTTTGAATAACAATATTACCATAAGCGAAAAACCTGATGGTTATATCTTAAATTTTGAAAATCTATACATGACTGACAATAAAAATTCATGTTTAACATCCAGTTTTTCTGGATTAGCATTATGTACTACAACTTTAAACAGACGATGTATGAGAGTCGAACATTTAACTTCATGTATCAAATCTTCTGTAGGTGACTATTACTGTGCACGATACATAATCGACTTTTTACCGTACATCCGATTCCATATCCGTAAAACAACCTGTTCCAACTTATATGATAAGTTTGATGTGCCTGATGGATTATACTCAGGACGAAATTTGGAGTTGATTGCACAACCTTGTCCAGGTTCCTCCAAAGAGCCGTTTAACCTAACTTTAATATCTGGTACGTACACAACTTTGCCATGTGGCTATACCTACGAAACAAATTATACATTCGAACCATTGCGTTTCTGCCGTTCTGACTGCGCTGTCGAATCTGTAGACGCTATCTATCACTACGTACCACCAATTATAACTGAAATAATTAAATCTATCAATACCTTAGACCGCTCATCTGTTGGTGTAAATACCCAAAGTTATCGAACTAATCTTTTAACAATTCAAAATCAAATAACTGAATACACCAATAACTTAACTAAAGATTATACCTATGATTTTAATACTTATTATAATCAATCTGAAGCTGATATAGTAACTGAAAAGTTGTCAGACTCAAAGGATATAATCGATAACATAAAAGTCACTACCCGGATATTGGACAAACAAATAGATTCTATACAAACCATCAGAGATGAGGGTGCGCATATTGGTCTATTAACCATTTTACATTTAGTATTAACTATGGTCCTTACAGGAGTAGTTATTACTATAAACAAACGATTACATAATCGTTATGACAAATTATCTATTTTGACCATAATATTACTTTTACCCGCTCTAGCTTTCGGTAAATTTACTAGTAATTGTACTTCACAATCTGAAGTTCAAGTATGCCGAATGGGTAAATATGATGCATGCCACCTCATAAATTCCACTAGCTATTGTTTTTGTGCTCCTACAAATAATCACACTATTTGGTCTGATTATGATGATTGTGTTGCACCAGGTCTTGCTATAACAACTTGGAATTCATTTAATAAACATATTTCACATGATATTAATTATCAAAACTTTTTCATATATCTTATATTGCTAATCGCAATTATTCAACTTATTTTATTCTACATATATGTAAGACCTATGTACTTTCGTAACTTCCCATCCGAACAAAACTTCGTTACCAATATACAATCAAAGTTTAAAAATCGAAAACGTAAATTACCTATTTTTAATAATAAAACTTTCTAAGTTGTTTGTTTCGGGTGGGCTATATATATTTTTAACAATTATAGATATTTTTATACAAATTATATCTTTTATTTACACAATAATGCAATCATTTACTACATTTTTATACATTGCGATAATAGTTTTATTACTTTATTTTATTTATCGCTTTTATCTATTGCTTAATATATTAAATCACATATTTTAACTATCACTTATAAAATTTATCTATCACTGGGTTTAAAATTATAAAATACGGTAGCTCTAATATACACTAATAATAATAATAATAATAAAAACTTTACTTTTTATAGACTAAAACTCGTCAGGACGAGGTTTTGTACCCAGAGTGCTTACCTTGACACCCACTTGTTCTATAATTAGTTGCTAGGGGTTTTGTTGCGACTTGCCTTAAACTAACATTCACTGAATCTTATTTTACGTCATTTATAATACACAAAGTTTTACCCACTGTCTTCTATAAGGCATAGTCCCTCAAATACCGCTATCATACTCCAAATAAATATAATTTGTTTGGTGTGTGGACATTATGAAGTAGCCGTAACAATAGTTGTATCTTGAGCTCCCGTA
